# Long subcutaneous tunnelling reduces infection rates in paediatric external ventricular drains

**DOI:** 10.1007/s00381-014-2523-3

**Published:** 2014-08-27

**Authors:** Christian D. E. Collins, John C. Hartley, Aabir Chakraborty, Dominic N. P. Thompson

**Affiliations:** 1 Southampton Medical School, University of Southampton, Southampton, UK; 2 Department of Microbiology, Level 4 Camelia Botnar Laboratories, Great Ormond Street Hospital for Children NHS Trust, Great Ormond Street, London, WC1N 3JH UK; 3 Department of Paediatric Neurosurgery, Great Ormond Street Hospital for Children NHS Trust, Great Ormond Street, London, WC1N 3JH UK; 4 Faculty of Medicine, Southampton General Hospital, University of Southampton, Mailpoint 801, South Academic Block Tremona Road, Southampton, SO16 6YD UK

**Keywords:** EVD, Ventricular drain, Infection, Hydrocephalus, Tunnelled, Ventriculostomy

## Abstract

**Purpose:**

The aim of this study is to report the efficacy of long subcutaneous tunnelling of external ventricular drains in reducing rates of infection and catheter displacement in a paediatric population.

**Methods:**

In children requiring external ventricular drainage, a long-tunnelled drain was placed and managed according to a locally agreed guideline. End points were novel CSF infection incurred during the time of drainage and re-operation to re-site displaced catheters. Data were compared to other published series.

**Results:**

One hundred eighty-one long-tunnelled external ventricular drains (LTEVDs) were inserted. The mean age was 6.6 years (range 0–15.5 years). Reasons for insertion included intraventricular haemorrhage (47 %), infection (27 %), tumour-related hydrocephalus (7.2 %), as a temporising measure (17 %) and trauma (2.2 %). The overall new infection rate for LTEVD was 2.76 %. If the 48 cases where LTEVDs were inserted to treat an existing infection are excluded, the infection rate was 3.8 % (5/133). The mean duration of insertion was 10 days (range 0–42 days). Four LTEVDs (2.2 %) were inadvertently dislodged, requiring reinsertion. Thirteen patients required removal of EVD alone.

There was a significant difference (*p* < 0.05) when comparing our infection rate to 14 publications of infection rates in short-tunnelled EVDs; however, there was no difference when comparing our data to three publications using LTEVDs.

**Conclusion:**

The use of an antibiotic-impregnated LTEVD, managed according to a predefined guideline, is associated with significantly reduced infection and displacement rates when compared with contemporary series. It is suggested that this reduction is of both clinical and economic benefits.

## Introduction

Insertion of external ventricular drains (EVDs) is one of the most common neurosurgical procedures performed in neurosurgery today, with over 20,000 EVDs inserted annually in the USA alone [1]. The most widely practised surgical technique for insertion of EVDs involves the insertion of a ventricular catheter into the ventricle with tunnelling of the distal end a short distance away from the incision, a *short-tunnelled* EVD.

The complication rate from short-tunnelled EVDs is high and includes infection, CSF leak, blockage, misplacement of the ventricular catheter at the time of surgery and the inadvertent migration of the ventricular catheter following surgery [2]. The overall complication rate ranges from 3.4 to 32.2 % [2–8].

The presumed mechanism of infection is bacterial entry at the exit site on the skin with subsequent ascending colonisation of the catheter. There are data suggesting that increasing the distance from the EVD exit site to the burr hole reduces infection [9–11].

We therefore hypothesised that insertion of a long-tunnelled EVD (LTEVD) would reduce the rate of *novel* infection and, since LTEVDs require the incorporation of a reservoir, would also reduce the rate of inadvertent migration of the catheter out of the ventricle.

We have defined a LTEVD as a ventricular access device comprising a ventricular catheter, a reservoir and a distal catheter that is externalised at the level of the abdomen or anterior chest wall. This paper is a retrospective audit of LTEVD insertions at a single paediatric neurosurgical institution, specifically addressing infection and mechanical complications.

## Methods

A retrospective review was conducted in all the patients who had a LTEVD inserted from 2 January 2008 to 9 March 2012. The policy of the neurosurgical department throughout the course of this study was to insert all EVDs in the operating theatre.

### Protocol

A local guideline for insertion of LTEVDs was in place for the duration of this study. This comprised antibiotic prophylaxis (flucloxacillin and amikacin) at induction of anaesthesia and for a further period of 24 h, clipping of hair at least 2 cm away from the wound and preparation of the field with povidone iodine solution and/or chlorhexidine (0.5 % in 70 % alcohol).

Bactiseal™ antibiotic-impregnated ventricular and peritoneal catheters were connected to a Miethke™ ventricular access device. Subcutaneous tunnelling was performed from the cranial wound to the chest wall or abdomen, allowing the distal catheter to be passed. A purse-string suture was applied to the exit site and covered with a waterproof dressing. The peritoneal catheter was not cut, allowing a long length of catheter to extend beyond the exit site.

Post-operatively, surgical and nursing staff followed the external ventricular drain clinical guideline of Great Ormond Street Hospital. Further details can be obtained from the lead author. Drain management involved hourly checks of the amount and colour of CSF drained, exit site condition and the patients’ neurological condition. Redness, inflammation, oozing of blood and CSF leakage at the exit site were all documented. The post-operative dressing was changed at 24 h using 2 % chlorhexidine Clinell™ wipes to clean the site. Following this, the dressing was changed weekly or when soiled.

Routine sampling of CSF from an EVD was not advocated unless there was a specific clinical indication for fear of introducing contamination into the sterile closed circuit. Patients presenting with shunt infection or de novo ventriculitis requiring EVD insertion had CSF sampling daily. For all other cases, CSF sampling was performed on the day prior to removal of the EVD or as part of a septic screen should the child demonstrate clinical evidence of infection. All CSF samples were sent to the on-site microbiology department and processed immediately. A cell count was made up of the neat CSF and a gram stain performed on the spun deposit, for a differential white cell count and for the presence of organisms. Primary culture of the spun deposit was performed on blood and chocolate agar incubated for 40–48 h at 35–37 °C in 5–10 % CO_2_, MacConkey agar incubated for 18–24 h in air at 37 °C, Sabouraud agar incubated for 5 days in air at 35–37 °C and blood agar incubated anaerobically for 5 days. Additional enrichment was performed by inoculation of brain-heart infusion for overnight enrichment at 35–37 °C followed by subculture on blood and chocolate incubated for 18–24 h.

EVD catheter tips were cultured by rolling the terminal 5 cm across a blood agar plate and incubating at 35–37 °C for 18–24 h.

### Data collection

In this study, a LTEVD infection was defined as a novel bacterial growth or detection in the CSF and/or LTEVD catheter that had arisen during the period of the LTEVD being in situ and that resulted in a change in treatment (antibiotic therapy and/or change of EVD). When assessing suspected cases of LTEVD infection, systemic factors, white cell count in the CSF, bacterial growth on culture from CSF/LTEVD catheter samples and antibiotic treatment were all considered before coming to a conclusion as to whether a LTEVD infection was present. LTEVD CSF infection was considered to have occurred if there was growth on primary culture, growth on subculture only and organisms were seen, growth in repeated samples on subculture when no organisms had been seen or where organisms were seen on repeated gram stain without growth. Growth from the tip without growth in the CSF was not automatically graded as a LTEVD CSF infection. Each case was managed on an individual basis. The lead microbiology consultant for neurosurgery and the neurosurgeon took the final decision as to whether the LTEVD had been infected during the management of the case and that decision has been used in this review.

The following data were retrieved from the departmental operative database, operating theatre log and clinical record.

The total number of LTEVD procedures performed during the study period, the number of LTEVD infections, the duration of insertion and the rate of inadvertent LTEVD dislodgement were recorded. LTEVDs require a further surgical procedure under general anaesthetic for removal. It was noted whether the removal of the LTEVD was part of another surgical procedure (such as insertion of ventriculoperitoneal (VP) shunt) or whether the removal was performed in isolation. Underlying diagnosis and patient age at the time of LTEVD insertion was also recorded.

Patients who required LTEVD insertion due to an existing infection were excluded from the data set when considering the final infection rate, due to possible treatment-induced discrepancies when testing for novel LTEVD infection.

When reporting the extra procedures needed for LTEVD removal, LTEVD insertions for the cohort with existing infection (*n* = 48) were omitted, as these patients would have had a subsequent procedure to reinsert a shunt in theatre, irrespective of the type of EVD inserted.

A 2 × 2 Fisher’s exact test and Student’s *t* test were used for assessing a significant difference between the categorical data.

## Results

One hundred seventy-seven patients had 181 LTEVDs placed between 2 January 2008 and 9 March 2012. The mean age of the patients was 6.6 years with a range of 0–15.5 years. Of the 181 procedures, 85 were inserted for intraventricular haemorrhage (47 %), 48 for infection (27 %), 13 for tumour-related hydrocephalus (7.2 %), 31 as a temporising measure (17 %) (where an EVD was inserted as an emergency measure and a definitive CSF diversion was performed subsequently) and 4 for trauma (2.2 %).

Of the 133 patients with no existing infection, microbiology analysis identified five cases of novel LTEVD infection, an infection rate of 3.8 %. No LTEVD infections were identified in the cohort (*n* = 48) that required a LTEVD due to an existing infection. Out of the total 181 procedures, five novel cases of LTEVD infection were identified, an infection rate of 2.76 %. Three other bacterial growths were recorded but were explained by sample contamination (two cases) and existing surgical site infection (one case).

As can be seen in Table 1, there was no obvious trend for infection in younger or older patients, with LTEVDs from a wide range of age groups being infected. When comparing the ages of the infected and non-infected groups, statistical analysis showed no evidence for age as a risk factor for LTEVD infections (*p* = 0.8967). Four of the five infected LTEVDs were inserted for longer than the median duration of insertion; however, there was no significant difference in LTEVD insertion duration between the infected (*n* = 5) and non-infected (*n* = 128) groups (*p* = 0.1881). One of the infected cases was inserted immediately after an endoscopic procedure. Four out of the five infected LTEVD cases were caused by coagulase-negative staphylococci (CONS).

**Table 1:** LTEVD infections in this series: an overview describing the organism found in the CSF, the age at LTEVD insertion, the duration of insertion of the LTEVD and the reason for insertion

Data set number	Organism on culture	Age at insertion	Duration of insertion (days)	Reason for insertion
145	Coagulase-negative staphylococcus	5 years and 11 months	7	A temporising measure (post endoscopic procedure)
169	Gram-positive cocci	9 months	28	Intraventricular haemorrhage
51	Coagulase-negative staphylococcus	15 years and 0 month	23	Intraventricular haemorrhage
88	Coagulase-negative staphylococcus	2 days	21	Temporising measure (spinal dysraphism)
79	Coagulase-negative staphylococcus	15 years and 4 months	12	Intraventricular haemorrhage

The mean duration of LTEVD insertion was 11.2 days (range 0 to 42 days and a median of 10 days, interquartile range of 19 days).

Four out of 181 (2.2 %) of the LTEVD procedures carried out during this time inadvertently dislodged, requiring an extra procedure to reinsert the EVD. No patients required EVD revision due to blockage. Thirteen extra procedures were carried out in theatre, specifically to remove the EVD, affecting 13/133 (9.8 %) of our patients (excluding the 48 LTEVDs inserted due to existing infection, as these would have an inevitable subsequent procedure to reinsert a non-infected shunt), where as the remainder of the removals were carried out during a subsequent procedure the patient required; thus, no extra procedures were needed to remove the EVD for these patients. The total number of extra operations required in this LTEVD cohort was thus 17 (9.4 %).

## Discussion

**Fig. 1:**
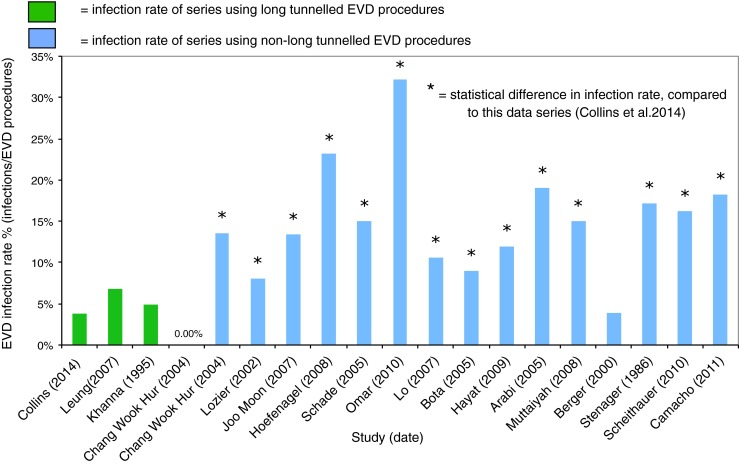
A comparison of EVD infection rates across previous series, using either a subcutaneous long-tunnelled EVD or a short/non-tunnelled procedure

A major complication of EVD insertion is CSF infection [2], posing a significant risk to patients as well as placing an additional burden on hospital resources. A range of infection rates from short-tunnelled EVDs has been reported, from 3.4 to 32.2 % [2–8, 12–20]. The data for infection rates of short-tunnelled EVDs are summarised in Fig. 1. This study, utilising LTEVD, reports a lower EVD infection rate when compared with previously published literature.

### EVD infections: the risk factors

Numerous studies have attempted to identify factors contributing to EVD-related infection. CSF leakage particularly near the catheter exit site [21, 22], duration of EVD insertion [3, 12], EVD reinsertion [23], presence [24] or absence [25] of prophylactic antibiotics and CSF sampling [5] have all been suggested as significant contributing factors towards the onset of EVD-related infection. Other factors such as breaches of the closed system [12] and surgical protocol violation [22] and regular catheter change [26] have also been investigated. Comparing existing series is fraught with difficulty due to the varying age groups studied (adult and paediatric), differing underlying aetiology and lack of standardised protocols for the insertion and management of EVDs. Currently, therefore, there is little agreement about the relative significance of these contributing factors towards CSF infection in EVD patients. What seems clear is that EVD infection is unacceptably high in most reported series. This exclusively paediatric study of long-tunnelled EVD incorporating a predefined guideline for perioperative EVD management has revealed an infection rate of 3.8 %, a rate substantially less than that previously reported.

### The evidence for tunnelling in reducing EVD infection

CSF leakage, either at the cranial site or the EVD exit site, is strongly correlated with infection risk. By increasing the distance from the burr hole to the exit site, the resistance to CSF flow around the outside of the catheter will be greater, thus reducing CSF leakage. Clearly, the distance that bacteria have to migrate in order to colonise the CSF compartment is also greater. Omar and Haspani found that tunnelling only as far as 5 cm from the burr hole was sufficient to reduce the infection rate from 62.9 to 11.5 % [9]. For postero-parietal or parieto-occipital burr holes in children, the options for *local tunnelling* are limited. We avoid exit sites in the region of the neck as this is a mobile area with multiple skin creases. Furthermore, wounds here are difficult to dress, are cosmetically unsatisfactory and may lie along the course of a subsequent shunt. Thus, we advocate tunnelling to the anterior abdominal wall or, less commonly, the anterior chest wall (well away from the nipple or breast bud), resulting in a tunnelling distance of at least 20 cm.

The results presented in this paper demonstrate comparable results to the other two studies [27, 28] that have investigated the effectiveness of LTEVDs. In the study by Khanna et al., a 0 % infection rate was achieved for the first 16 days after LTEVD insertion and an overall infection rate of 4 %, in 100 adult and paediatric patients [27]. It was concluded that LTEVDs had a low rate of infection and could remain in place for up to 40 days. A more recent study [28], using a non-antibiotic-impregnated LTEVD, in 114 adult and paediatric patients requiring more than 7 days drainage, found the infection rate to be 6.8 %, which was deemed comparable with the infection rate of standard short-tunnelled EVDs. Notably, LTEVDS were capable of remaining in place for long periods of time (up to 60 days, with a mean duration of 20 days) and no additional morbidity was associated with the LTEVD procedure.

Both of these studies indicate promise of the LTEVD technique though without conclusive evidence of their effectiveness in the paediatric population.

### Further strengthening the LTEVD argument: experience from this single-centre study

Our study demonstrated a LTEVD infection rate of 3.8 % (five out of 133), which, as demonstrated by Fig. 1, compares favourably with previously published data on non-LTEVD series. A review of EVD infection [2], using data from 23 studies, comprising 5,733 EVD procedures in both adults and children, reported an infection rate of 8.8 %. Figure 1 compares the infection rates in 15 publications [2, 3, 8, 9, 11, 19, 20, 23, 29–35] using short-tunnelled EVDs and 3 publications using LTEVDs [27–29] with our series. A statistically significant difference (*p* = <0.05) was found in the EVD-related infection rate between our study and 14 of the studies using the short-tunnelled procedure. Furthermore, there was no significant difference in the infection rates when comparing our study to the other three published studies using a long subcutaneous tunnel, suggesting that tunnelling seems to be a major contributing factor when considering EVD infection.

The infection rate reported does not include the 48 cases that required a LTEVD insertion for an existing infection as these patients may have been on active treatment for existing infection, which may affect the ability to detect a novel infection. Infection is a well-recognised complication of EVD insertion in patients with *clean* CSF before insertion. However, there is also a rate of novel infection, with new organisms, in the group where the EVD is placed as a part of the treatment for infection. There were no reported novel EVD infections in this group with an existing infection (*n* = 48). Therefore, the total infection rate, including this group that require a LTEVD for infection, is 2.76 % (five out of 181).

Four out of 181 (2.2 %) LTEVDs in this study were inadvertently dislodged and required a separate procedure to reinsert the EVD. Data on the rate of displacement of EVD are sparse. In another study, using short-tunnelled EVD, a dislodgement rate of two out of 51 (8.1 %) was reported [11]. This would imply that the combination of a reservoir and LTEVD affords some protection against this complication. The average duration of insertion was 11.2 days, with one LTEVD remaining in place for 42 days. Our data and the data of the other LTEVD studies [27–29] suggest that LTEVDs can safely remain in place for long periods of time without the attendant risks of infection and dislodgement. It was noted that four of the five infected LTEVDs were inserted for longer than the median duration of insertion; however, a statistical comparison found no significant difference in the ages of the two groups. Duration of EVD insertion has been shown to be a significant risk factor when using short-tunnelled EVDs [2, 3], further strengthening the argument for a LTEVD protocol.

Additionally, one of the infected cases was inserted immediately after an endoscopic procedure, suggesting that this may have been a contributory factor to the LTEVD infection, which would concur with the previous literature where intraoperative endoscope use was considered to be an additional risk factor when considering shunt infection rates in the paediatric population [36].

The use of a LTEVD incorporating a reservoir will inevitably result in an extra surgical procedure (to remove the EVD) in some children. This study reported an extra procedure rate of 9.4 %. We would argue that the benefits of the long tunnel in terms of reducing infection rate and inadvertent dislodgement outweigh this inconvenience.

### Antibiotic-impregnated catheters: their importance in reducing EVD infections

The importance of using an antibiotic-impregnated catheter (AIC) to lower infection rates in this cohort must not be underestimated. The Bactiseal™ catheters used for this cohort contained 0.15 % clindamycin and 0.054 % rifampicin. Several recent studies have demonstrated the effectiveness of using antibiotic-impregnated EVD catheters in both the adult and paediatric population. A recent systematic review and meta-analysis [37] identified several studies in both the adult and paediatric populations where antibiotic impregnation of the EVD catheter was beneficial in reducing EVD-related infections.

A six-centre randomised controlled trial in the adult population [38] has also shown a significant reduction in LTEVD infections, from 9.4 to 1.3 % when using minocycline- and rifampicin-impregnated catheters [38]. In a prospective versus historical cohort study of 91 paediatric patients, Tamburrini et al. [39] reported a significant reduction in EVD infection rates, from 31.8 to 2.1 % when using AICs in paediatric EVDs. In a further study, when AICs were inserted in a centre already using prophylactic, systemic antibiotic treatment, EVD infection rates were reduced from 23.5 to 4.3 % [40], further strengthening the argument for AIC use in EVDs.

When considering the antibiotics impregnated into the catheters, both minocycline/rifampin-impregnated ventricular catheter and clindamycin/rifampin-impregnated EVD catheter (used in this study) have been shown to be equally effective at reducing EVD-related infections [41].

However, not all of the data suggest that AICs have a beneficial impact in EVD insertion. A randomised controlled trial of 184 patients from another centre [42] showed that AICs did not have a significantly higher rate of CSF infection (1 %) compared to catheters without antibiotic impregnation (3 %), and there was no significant difference of clinical outcome at 6 months. A more recent randomised controlled study also demonstrated similar findings in 348 adult patients, where AICs used in EVDs did not contribute to a reduction in infection rate; however, this could be attributed to a low baseline rate of infection and so difficult to prove an AIC-related reduction [43].

The risk of a false negative CSF culture when drawing CSF from an AIC must also be considered. Using an in vitro model, an increased risk of false negative results of infected CSF has been demonstrated [44]. Additionally, an increased rate of infection has been noted in paediatric VP shunt patients with prior EVD insertion using an AIC [45], the authors postulated that prior use of an AIC increased the potential for resistant organisms.

In summary, despite the possible risk of false negative CSF sampling and antibiotic resistance, there is a body of both level I and level II evidences, supporting the conclusion that AICs do reduce EVD infection rates.

The role of AICs used as a part of the treatment protocol for patients with pre-existing infection has been debated [46]. However, for the cohort requiring LTEVD insertion for pre-existing infection in this study (*n* = 48), the AIC tubing was not employed as a specific treatment for the infection, for which appropriate intravenous and intraventricular antibiotics were administered before LTEVD insertion. Therefore, despite the licence for Bactiseal™ AIC catheters being for prevention of infection only, it was departmental practice to use AICs for all EVD insertions, whether infection was suspected at insertion or not.

The relative contribution of AICs, a long subcutaneous tunnel and a pre-defined protocol for perioperative management of the EVD is difficult to elucidate in this study. There is evidence from another study that a comprehensive EVD management protocol, similar to that implemented in this cohort, was effective in reducing EVD-related infection rates. Kubilay et al. cited a reduction in infection rate from 1.06 to 0 % in EVD procedures, over a 4-year period [47]. The effectiveness of protocols in reducing shunt infection has also been well demonstrated in the North American Shunt registry [48].

### Implications and limitations

This low infection rate and low dislodgement rate shown for EVDs in this study, using a LTEVD, an AIC and a perioperative management protocol, represents a potential for both clinical and economic benefits. Fewer EVD infections would reduce the patient’s duration of hospital admission and reduce the risk from further surgery required to replace an infected EVD. A lower dislodgement rate is both safer for the patient and cost-effective, as there is a reduced risk and cost brought about by extra theatre time.

Our study does have some limitations. The multi-study comparison (Fig. 1) does not take into account the different operating protocols and infection definitions across the centres at which the procedures were carried out. As discussed, EVD-related infection is a multi-factorial problem [49] and it would be difficult to disregard all the compounding factors apart from the tunnelling length and the use of AICs that may influence the comparison with the other studies. This study was also conducted in the paediatric population, and comparing with adult studies may not offer a true comparison. Further studies may wish to assess the cost-effectiveness of this procedure, specifically in relation to hospital stay and extra operating time.

## Conclusion

Long-tunnelled EVDs, using an antibiotic-impregnated catheter and managed according to an agreed perioperative management protocol, carry a lower infection rate and dislodgement rate than the more widely used short-tunnelled procedure. We believe that the reduced morbidity and costs associated with such a policy outweigh the disadvantage of requiring an additional procedure for EVD removal.
